# Effects of Short-Term Intake of Montmorency Tart Cherry Juice on Sleep Quality after Intermittent Exercise in Elite Female Field Hockey Players: A Randomized Controlled Trial

**DOI:** 10.3390/ijerph191610272

**Published:** 2022-08-18

**Authors:** Jinwook Chung, Minkyung Choi, Kihyuk Lee

**Affiliations:** 1 Department of Sport Culture, Dongguk University, 30, Pildong-ro 1gil, Jung-gu, Seoul 04620, Korea; 2 Department of Sports Science Convergence, Dongguk University, 30, Pildong-ro 1gil, Jung-gu, Seoul 04620, Korea

**Keywords:** tart cherry juice, recovery, melatonin, cortisol, sleep quality, actigraphy, female field hockey players

## Abstract

The purpose of this study was to investigate the effect of short-term consumption of tart cherry juice on levels of cortisol and melatonin and sleep quality after intermittent exhaustion exercise in female elite field hockey players. A total of 19 field hockey players participated in the present study for 5 days. The individuals were divided into the placebo group (PLA, *n* = 9) and the tart cherry juice group (TCJ, *n* = 10), respectively. Actigraphy devices were distributed to analyze sleep quality and participants were required to wear the device while sleeping until the study was completed. Participants consumed tart cherry juice or placebo drinks five times in a total of 48 h while double-blinded after intermittent exhaustion exercise. A significant interaction effect (group × time) between PLA and TCJ groups was not observed in the levels of melatonin and cortisol. The variables of sleep quality showed significant interaction effects with regards to the total time in bed (TTB; *p* = 0.015), wake after sleep onset (WASO; *p* = 0.044), and movement index (MI; *p* = 0.031) variables. As a result, our study confirmed the possibility that the short-term intake of tart cherry juice could not change the levels of melatonin and cortisol in elite female hockey players but could help improve their sleep quality.

## 1. Introduction

The cherry is a fruit with a relatively low number of calories and numerous significant nutrients, including vitamins A, C, and polyphenol [1]. Cherry varieties are mainly divided into sweet (*Prunus avium*) and sour varieties (*Prunus cerasus*), and the representative variety of sour cherries is the (Montmorency) tart cherry [2]. Tart cherry was demonstrated to have a relatively higher content of anthocyanin and phytochemicals than other fruits and induced antioxidative and anti-inflammatory effects [3,4]. The intake of tart cherry juice revealed a positive effect on the improvement in the levels of indicators (Creatine Kinase, Interleukin-6, and Interleukin-8) after exercise-induced muscle damage that resulted in inflammation or in the generation of fatigue substances [5,6]. Research on the positive effects of cherry juice intake in athletes has been conducted in various fields of sports. Previous studies reported the positive effects of consumption of tart cherry juice on recovery in cyclists, soccer players, and marathoners [7,8,9]. In addition, a study on healthy people reported that consumption of tart cherry juice can quickly promote sleep, cognition, and a decrease in the level of oxidative stress and training-related muscle damage [10,11].

Interestingly, tart cherry contains high concentrations of melatonin [12]. Melatonin is a substance that strongly affects the human sleep–wake cycle and nocturnal core temperature to induce sleep, and physiologically, the secretion of melatonin in the human body is controlled according to the light–dark cycle [13,14]. It has previously been reported that the intake of tart cherry juice helps to improve sleep quality in people with late-life insomnia; however, the study only confirmed subjective improvement in sleep quality [15]. In another study, tart cherry juice intake in healthy people showed a significant increase in the amount of melatonin in urine compared to the control group and a significant increase in the efficiency of objective sleep using an actigraphy device [16]. As such, studies on improving the quality of sleep after the intake of tart cherry juice have included people with insomnia and healthy people. However, the effect of tart cherry juice on sleep quality has not been sufficiently elucidated in the case of athletes.

Field hockey is a 15-min four-quarter event that runs for a total of 60 min and is an intermittent team sport characterized by acceleration, deceleration, and direction change [17]. Field hockey is a sport that requires a high level of physical strength and fast speed to move short distances in a repeated manner [18]. Previous studies have reported frequent injuries, muscle damage, and inflammation due to intensive schedules that require up to three games for four consecutive days for field hockey international competitions [19]. The injuries and inflammation that are succumbed to during the game undermine the quality of sleep in players and hinder a quick recovery [20,21]. The current schedule of a field hockey tournament requires playing the next game even when the players have not recovered. Nevertheless, to win the match, it is important to maintain performance during the game through rapid recovery; hence, rapid recovery through improving sleep quality should be achieved [22].

To date, no studies have demonstrated the effect of short-term tart cherry juice intake on sleep quality in field hockey players who need a quick recovery due to their game schedule. Especially, there is a lack of studies analyzing the effects of tart cherry juice intake in female athletes with lower recovery levels than male athletes [23,24,25]. The purpose of this study was to examine the effect of the intake of tart cherry juice on the levels of melatonin, cortisol, and the quality of sleep after intermittent exhaustion exercise in female elite field hockey players.

## 2. Materials and Methods

### 2.1. Participants

**Figure 1:**
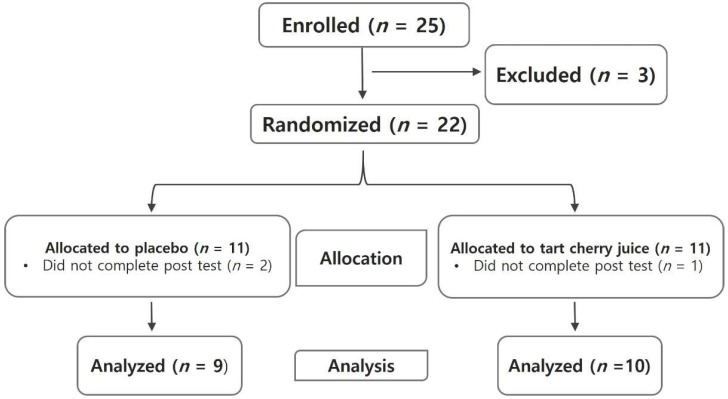
Flow chart of the study.

The participants of this study were elite women’s field hockey players at H University. The sample size was estimated using the G*Power version 3.1 (Franz Faul, Kiel, Germany) under the following conditions: effect size, 0.28; alpha-error, 0.05; power, 0.70. The power calculation results show that 11 participants were sufficient for each group. A total of 22 people participated in this study. Finally, data of 19 participants were used for the analysis. The flowchart of this study is shown in Figure 1. The participants were sufficiently briefed about this study procedure, potential risks, and precautions before signing the prior consent form. It was confirmed whether there was an allergic reaction due to fruit intake including cherry juice. As a result, no participants ever experienced a fruit allergy reaction, and no one experienced fruit allergy in our study. The study involved the athletes who expressed their intention to participate. The criteria for selecting subjects were elite field hockey players with more than 6 years of experience and no injuries for more than 6 months based on the research data. Research approval was approved by the research ethics committee of the university (DUIRB-202109-05). The physical characteristics of the participants are shown in Table 1.

### 2.2. Procedures

**Figure 2:**
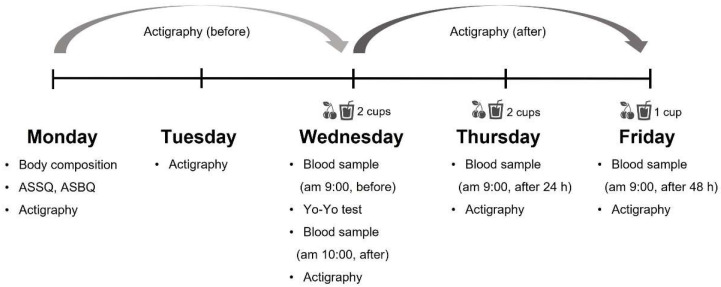
A brief design of the study.

To examine the effect of tart cherry juice intake, two groups were designed, the tart cherry juice intake group (TCJ) and the placebo group (PLA), to ensure double blindness. The participants were randomly assigned to each group. The final participants were TCJ (*n* = 10) vs. PLA (*n* = 9). In previous studies, the TCJ intake period was set from 7 to 10 days to investigate the effect of TCJ intake on recovery in elite athletes [24,26,27]. However, in this study, a short period of 48 h was set as the TCJ intake period. This study controlled for those factors other (nutrition and intensity of training) than tart cherry juice, so that they would not affect the experiment as much as possible by conducting a camp training period in which participants consumed the same food and moved according to the same schedule, and experiments were conducted twice with each protocol. On the first day of the experiment, the participants were notified of precautions to be taken during the study period, and height and weight were measured. Actigraphy devices were distributed for sleep quality analysis and were advised to be worn by the participants during bedtime. Meanwhile, the athlete sleep behavior questionnaire (ASBQ) and the athlete sleep screening questionnaire (ASSQ) were administered to evaluate the sleep quality level of the subjects on the first day [28,29]. The evaluation revealed no significant difference in sleep quality between the two groups; see Table 1. On the second day of the experiment, all the participants performed the same schedule and wore the actigraphy device at bedtime to analyze the quality of sleep. On the third day of the experiment, participants’ venous blood samples were collected during stabilization. The standardized warm-up was performed before the intermittent exhaustion exercise. Subsequently, the Yo-Yo intermittent recovery test was conducted to analyze the recovery reaction after intermittent exercise to induce exhaustion. After intermittent exhaustion exercise, venous blood was immediately collected, and written instructions such as how to consume tart cherry juice and precautions were provided to the participants. On the 4th day of the experiment, venous blood was collected at 9 a.m. Finally, on the 5th day of the experiment, venous blood was collected at 9 a.m. The actigraphy device was collected to analyze the quality of sleep. A brief design of this study is presented in Figure 2.

### 2.3. Intake of Tart Cherry Juice

The participants were divided into the tart cherry juice group (TCJ) and the placebo group (PLA). The subjects were advised to consume the tart cherry concentrate (Organicmaru, Agricultural Co., Ltd., Gwangju, Korea) or placebo beverage 5 times in a total of 48 h while being double-blinded. The tart cherry concentrate was diluted with water, such that each 30 mL serving was made up into a 200 mL beverage [9,27]. According to the manufacturer, 30 mL of tart cherry concentrate contains about 48 tart cherries produced in Turkey. A previous study reported that tart cherry concentrate contains a total of 213 ± 41 μg/g anthocyanin [30]. In the PLA group, the participants consumed beverages mixed with water and vinegar, so that the color and taste were similar to the tart cherry concentrate diluted in water. The participants in both of the groups consumed 200 mL of the beverage in the morning and 200 mL in the evening on the first and second days and 200 mL in the morning on the third day.

### 2.4. Intermittent Exhaustion Exercise

Intermittent exhaustion exercise was performed using Yo-Yo intermittent recovery test 1. During the test, when a beep was heard from the starting line (B), the distance of 20 m from the cone (C) in front had to be reached before the next beep, and then the participant had to return to the starting line (B). After returning, the participant was required to jog for 10 s with the red cone (A) in the front and then recover. The participant had to reach the specified departure line and arrival line section according to the signal, and if the specified section was not reached, one warning was given, and the second round was eliminated. The Yo-Yo intermittent recovery test 1 in this study is a method conducted to induce fatigue in the participants, and all the participants underwent the test up to a minimum of stage 19.

### 2.5. Blood Analysis

For stabilization, all the participants rested for 30 min before blood collection, and about 10 mL of blood was collected from the forearm vein with a disposable syringe. Blood analysis was performed 4 times in total before, after, after 24 h, and after 48 h. The collected blood was stored at 4 °C and plasma was separated at 3000 rpm for 15 min using a centrifuge (Labogen 1248R, BMS., Seoul, Korea) and immediately stored at −80 °C. Subsequently, the levels of melatonin and cortisol in the blood were analyzed according to the intake of tart cherry juice.

### 2.6. Actigraphy Analysis

All the participants wore an actigraphy device for 5 days (4 nights) from Monday to Friday for the measurement of variables related to sleep quality. Precautions were conveyed before participants wore the actigraphy device, and participants were instructed to secure a wristwatch, actigraph wGT3X-BT (ActiGraph LLC., Pensacola, FL, USA), around the non-dominant wrist before going to bed so their quality of sleep could be evaluated. The quality of sleep was analyzed by comparing the time of sleep and wake-up every night with the activity data. The following objective sleep parameters were collected: sleep efficiency (SE), total time in bed (TTB), total sleep time (TST), wake after sleep onset (WASO), number of awakenings (NOA), average awakening length (AAL), movement index (MI), and fragmentation index (FI). Raw active data stored as digitized computer data were analyzed by ActiLife version 6.9.2 software (ActiGraph, LLC., Pensacola, FL, USA), and the accelerometer of the actigraph was initialized to collect data and automatically score power saving at 80 Hz every 60 s [31]. The average value was calculated using the Monday and Tuesday data before intermittent exhaustion exercise as the data before intervention during the 5 days (4 nights), and the average value was calculated using the Wednesday and Thursday data after exercise as the data after the intervention.

### 2.7. Statistical Analysis

The WIN/SPSS program (SPSS, Inc., Chicago, IL, USA), version 25.0, was used for the values of all the variables measured in this study. The measured values were calculated as the mean, standard deviation, and standard error. A two-way repeated measures ANOVA was conducted to find out the differences in blood variables (melatonin, cortisol) and sleep quality variables according to the intake of tart cherry juice and the placebo. When the interaction was observed, the rate of change in the values of blood variables between the groups was analyzed using an independent t-test. As for the quality variables of sleep, an independent t-test was performed between the groups, and a paired t-test was performed before and after. Significance was set at α = 0.05. The effect size (eta-squared) for the two-way ANOVA interpreted 0.01, 0.06, and 0.14 as small, medium, and large, respectively.

## 3. Results

### 3.1. Changes in the Blood Variables According to the Intake of Tart Cherry Juice

The changes in the levels of serum melatonin and cortisol according to the intake of tart cherry juice are shown in Table 2. The levels of melatonin and cortisol relative to the to the intake of tart cherry juice five times over 48 h showed no significant interaction effects (group × time) between the PLA group and the TCJ group.

### 3.2. Changes in the Quality of Sleep According to the Intake of Tart Cherry Juice

Table 3 shows the changes in sleep quality variables according to the intake of tart cherry juice by the participants. The variables of sleep quality relative to the intake of tart cherry juice five times over 48 h showed significant interaction effects (group × time) with regards to the TTB (*p* = 0.015), WASO (*p* = 0.044), and MI (*p* = 0.031) variables. In addition, TTB showed significant differences (*p* = 0.005) before and after in the TCJ group and significant differences (*p* = 0.005) between the PLA group and the TCJ group after the intake of tart cherry juice. In the case of WASO, it showed significant differences (*p* = 0.024) before and after in the TCJ group.

## 4. Discussion

The purpose of this study was to examine the effect of the intake of tart cherry juice (TCJ) five times on melatonin and cortisol levels and sleep quality during 48 h after intermittent exhaustion exercise in female elite field hockey players. We assumed that the level of melatonin would increase, cortisol would decrease, and consequently there would be an improvement in sleep quality after TCJ consumption. Our study is the first to prove that the intake of TCJ by elite female field hockey players improves sleep quality, although it did not affect changes in the levels of melatonin and cortisol.

A previous study reported that the intake of TCJ improved the quality of sleep in the elderly with moderate/severe insomnia due to an increase in the exogenous melatonin content provided by the tart cherry [15]. However, the melatonin level was not measured, and sleep quality was analyzed through a subjective questionnaire. Meanwhile, the results of another study provide additional evidence that improvement in sleep quality was mediated by an increase in the dietary melatonin present in tart cherries. The researchers reported that the intake of TCJ significantly increased the content of melatonin in urine in healthy adults [16]. They reported that the intake of TCJ for seven days without any intervention induced an increase in the melatonin levels in urine. The secretion of melatonin was affected by the light–dark cycle and eventually played an important role in the regulation of the sleep–wake cycle [13]. Concerning the fact that endogenous melatonin affected the core temperature and facilitated sleep [14], it was hypothesized that increased levels of exogenous melatonin would further promote changes in the core temperature, thereby contributing to improved sleep quality. However, the previous study was inconsistent with our results, which state that the intake of TCJ by female field hockey athletes after intermittent exhaustion exercise did not significantly change the levels of melatonin. Variables such as serum melatonin and sleep quality might differ from individual to individual. Moreover, it should be considered that the half-life of melatonin is relatively short and the fluctuation in melatonin levels may be missed throughout the day. Furthermore, considering that sleep control is affected by pro-inflammatory cytokines [32], the anti-inflammatory and antioxidative characteristics of tart cherries might have induced control over the sleep index in this study due to the presence of numerous phenolic compounds [7,33].

Meanwhile, we investigated the effect of the intake of TCJ on the level of serum cortisol, a stress marker, after a short period of intermittent exhaustion exercise. Immediately after intermittent exhaustion exercise, the secretion of cortisol decreased in both of the groups but returned to its original state 24 h and 48 h after exercise, and there was no significant difference between the two groups. Previous studies reported that the intake of cherry powder supplements by young men for 10 days led to a decrease in cortisol levels as compared to the control group 48 h after exercise [24]. However, since in our study the participants consumed only five portions of TCJ in 48 h, it is believed that short-term consumption may not help to reduce cortisol levels after exhaustion exercise. Although the results may vary depending on the individual’s condition.

To evaluate the quality of sleep due to TCJ intake, participants were asked to wear a wristwatch-type actigraphy device during the experimental period, and data collected from the actigraphy device were analyzed. TTB, WASO, and MI showed a significant interaction between the groups and the pre–post period among other variables in the actigraphy that measure sleep quality. Moreover, there was no significant difference TST between the TCJ group and the PLA group, but it is noteworthy that significantly positive results were found in the TTB, indicating total time in bed, WASO, indicating time of wake after sleep onset, and MI, indicating movement during sleep. In a previous study, consumption of TCJ for seven days in healthy adults led to a significant increase in TTB and TST [16]; however, TTS did not show any significant difference in the present study. Although the time spent lying in bed had decreased, it indicated an increase in the time spent sleeping and could have affected the quality of sleep. Our study was conducted during the camp training period during which the participants’ bedtime and waking hours were fixed and they were active by following the same schedule.

Based on our results, it is proposed that the intake of TCJ improves sleep quality in athletes with a tight game schedule and can help them recover from injuries and inflammation that are succumbed to during the game. Numerous studies have pointed out that many players suffer from insomnia due to fatigue, slow recovery, and stress ahead of the game, or that the quality of sleep is low [34,35,36,37]. We already reported in a previous study that the five-time intake of TCJ over 48 h after intermittent exercise affects the reduction of IL-6 [38], an indicator of inflammation in the results. Moreover, Bell et al. (2016) reported that the intake of TCJ after intermittent exercise significantly reduced muscle pain as well as IL-6 [9]. Therefore, although a significant increase in melatonin could not be observed in this study, the decrease in Il-6 and muscle pain seemed to have a positive effect on the quality index of sleep of subjects. In particular, WASO and MI, which confirmed significant effects in this study, are indicators related to awakening during sleep, and it seems that the reduced Il-6 and relieved muscle pain may have affected these sleep quality variables. Considering the results of the present study, it is proposed that the intake of TCJ during the game season or tournament schedule is a valid strategy to recover from fatigue and improve sleep quality.

A reported study conjectured that the positive effect of tart cherry intake on sleep may lead to improvement in circadian regulation [15]. Therefore, it is believed that the intake of TCJ can help control the circadian rhythm in people with sleep disorders. The clear difference between this study and previous studies lies in the selection of the subject group. In previous studies, the elderly, suffering from insomnia, or healthy adults were targeted [15,16]. Our study was conducted on athletes who may have difficulty controlling their condition due to intense exercise. Nevertheless, the results reveal that the short-term intake of TCJ improved variables that represent sleep quality.

There were several limitations to our study. First, since all the participants in our study were women, we could not rule out that they were at different stages of the menstrual cycle, which can also affect the deep temperature and sleep disorder propensity [39]. However, the schedule of the experiment was adjusted in consideration of the menstrual cycle. No participants experienced menstruation during the experiment. Second, we should consider the possibility that external factors might also have influenced the study results, although, in the present study, we targeted athletes during camp training to minimize the impact of other factors. Moreover, the intake of TCJ five times in 48 h is a relatively short period. Therefore, in the future, further studies should prove the improvement in sleep quality due to TCJ intake by considering the above-mentioned drawbacks.

## 5. Conclusions

Our study is the first to demonstrate that short-term TCJ intake can improve sleep quality in elite female hockey players. Although the intervention of other factors cannot be completely excluded, our results support the results of previous studies that demonstrated an improvement in sleep quality through TCJ intake. Consequently, it is proposed that TCJ can help female athletes’ quick recovery for future matches.

## Figures and Tables

**Table 1:** Physical characteristics of the participants.

Variables	Age (Years)	Height (cm)	Weight (kg)	Career (Years)	ASBQ (Score)	ASSQ (Score)
PLA	21.78 ± 1.39	162.11 ± 7.74	56.78 ± 9.85	7.67 ± 1.41	40.89 ± 3.62	11.33 ± 2.69
TCJ	21.30 ± 1.06	162.20 ± 5.87	57.30 ± 3.77	7.10 ± 1.29	38.60 ± 7.02	11.50 ± 2.59

PLA, placebo group; TCJ, tart cherry juice group; ASBQ, athlete sleep behavior questionnaire; ASSQ, athlete sleep screening questionnaire, values are the means and standard deviations.

**Table 2:** Change in melatonin and cortisol levels according to the intake of tart cherry juice.

Variables	Group	Before	After	After 24 h	After 48 h		F	*p*	ES
Melatonin (pg/mL)	PLA	21.81 ± 14.96	19.77 ± 18.49	24.49 ± 27.75	21.45 ± 16.11	G T G × T	0.011 2.985 1.365	0.918 0.040 0.264	0.001 0.149 0.074
	TCJ	23.06 ± 12.49	19.23 ± 11.18	22.67 ± 12.19	25.61 ± 14.82				
Cortisol (μg/dL)	PLA	15.21 ± 2.80	9.60 ± 2.09	15.93 ± 4.78	12.50 ± 3.19	G T G × T	2.775 28.512 0.524	0.114 0.000 0.668	0.140 0.626 0.030
	TCJ	17.35 ± 3.16	11.59 ± 2.61	17.47 ± 4.48	15.81 ± 4.15				

PLA, placebo group; TCJ, tart cherry juice group; G: group, T: time, G × T: group × time; ES, effect size; values are the means and standard deviations.

**Table 3:** Change in sleep quality variables as per actigraphy.

Variables	Group	Before	After		F	*p*	ES
SE (%)	PLA	85.53 ± 7.48	82.20 ± 6.99	G T G × T	1.027 0.374 4.054	0.325 0.549 0.060	0.057 0.022 0.193
	TCJ	85.37 ± 8.98	87.68 ± 7.00				
TTB (min)	PLA	429.39 ± 37.37	414.38 ± 34.94	G T G × T	0.001 13.097 7.380	0.970 0.002 0.015	0.000 0.435 0.303
	TCJ	473.70 ± 102.38	368.40 ± 24.25 ^A,B^				
TST (min)	PLA	368.44 ± 52.93	337.17 ± 47.54	G T G × T	0.239 12.411 2.494	0.631 0.003 0.133	0.014 0.422 0.128
	TCJ	337.17 ± 47.54	322.35 ± 32.67				
WASO (min)	PLA	54.06 ± 28.78	66.39 ± 29.95	G T G × T	0.259 0.246 4.748	0.618 0.626 0.044	0.015 0.014 0.218
	TCJ	63.30 ± 44.40	43.70 ± 24.13 ^C^				
NOA (no/night)	PLA	19.06 ± 7.73	17.72 ± 6.25	G T G × T	0.194 6.442 1.979	0.665 0.021 0.177	0.011 0.275 0.104
	TCJ	19.60 ± 5.53	14.95 ± 4.56				
AAL (index)	PLA	2.80 ± 0.82	4.63 ± 4.21	G T G × T	2.129 1.502 2.129	0.163 0.237 0.163	0.111 0.081 0.111
	TCJ	3.05 ± 1.47	2.90 ± 1.84				
MI (index)	PLA	14.78 ± 4.01	18.67 ± 5.95	G T G × T	0.280 0.724 5.563	0.603 0.407 0.031	0.016 0.041 0.247
	TCJ	16.31 ± 7.70	14.49 ± 5.85				
FI (index)	PLA	15.27 ± 8.53	12.29 ± 4.78	G T G × T	2.084 2.485 0.134	0.167 0.133 0.719	0.109 0.128 0.008
	TCJ	12.85 ± 5.40	8.08 ± 9.39				

PLA, placebo group; TCJ, tart cherry juice group; SE, sleep efficiency; TTB, total time in bed; TST, total sleep time; WASO, wake after sleep onset; NOA, number of awakenings; AAL, average awakening length; MI, movement index; FI, fragmentation index; MI, movement index; G, Group; T, Time; G × T, group × time; ES, effect size; values are the means and standard deviations; ^A^, significant differences (*p* = 0.005) with PLA; ^B^, significant differences (*p* = 0.005) before; ^C^, significant differences (*p* = 0.024) with PLA.

## Data Availability

Not applicable.
